# Awareness of psychiatrists regarding physician suicide and prevention in developed and less developed countries

**DOI:** 10.1192/j.eurpsy.2024.397

**Published:** 2024-08-27

**Authors:** K. Kostyál, J. H. Terje, P. Z. Álmos

**Affiliations:** ^1^Department of Psychiatry, Albert Szent-Györgyi Medical School, University of Szeged, Szeged, Hungary

## Abstract

**Introduction:**

The World Health Organization estimates that more than 700,000 people worldwide die by suicide every year. Suicide is a complex issue, and occupation can be considered one of the risk factors. Data from the USA indicates that the suicide rate among doctors is higher compared to the general population. Among different specialties, general practitioners face the highest risk, followed by internal medicine, and then psychiatry. Apart from Anglo-Saxon countries, available data regarding physician suicide is limited; in some countries, the topic is considered taboo.

**Objectives:**

The goal of this pilot study was to explore whether psychiatrists in different countries have access to suicide databases that include occupational information and to determine what prevention strategies and interventions are currently in use.

**Methods:**

We distributed a short questionnaire to a group of psychiatrists (n=25) to assess the existing methods in their respective countries for collecting suicide data and implementing suicide prevention measures. The survey included both developed and less developed countries. Out of the 20 participating countries, 12 returned our questionnaire by the deadline. The final participating countries were Croatia, Czech Republic, Ethiopia, France, Germany, Hungary, Kazakhstan, Mexico, Qatar, Serbia, Sweden, and the UK.

**Results:**

Based on our colleagues’ reports, none of the responding countries have publicly available data on the number of physicians who committed suicide in the last three years. The risk of suicide and substance abuse among doctors is not systematically assessed or published in any of the participating countries. Kazakhstan is the only country where burnout, anxiety, and depression among doctors are regularly assessed. Ethiopia is the only participating country without a hotline for individuals in a suicide crisis. Mexico, Qatar, and Kazakhstan are the only countries with dedicated hotlines for health workers. Regarding preventive strategies, colleagues from Hungary, Serbia, Sweden, and Ethiopia did not report any strategies specifically aimed at preventing physician suicides. Germany and the UK were the two countries with more than one prevention strategy, both providing a free toolkit to identify and support at-risk populations. There are significant differences in the amount of mental health support that doctors receive in each country.

**Conclusions:**

Psychiatrists are not aware of physician suicide data and the utilization of preventive strategies vary widely among the participating countries. There is no standard practice for screening doctors for suicide risk, burnout, anxiety, depression, substance abuse, or adequate data collection on suicide. Based on these findings, it would be necessary to include more countries in the sample and conduct a more detailed examination of the issue in the future.

**Disclosure of Interest:**

None Declared

